# Tendon proper- and peritenon-derived progenitor cells have unique tenogenic properties

**DOI:** 10.1186/scrt475

**Published:** 2014-07-08

**Authors:** Michael J Mienaltowski, Sheila M Adams, David E Birk

**Affiliations:** 1 Department of Molecular Pharmacology & Physiology, University of South Florida, Morsani College of Medicine, 12901 Bruce B. Downs Blvd, MDC 8, Tampa, FL 33612, USA; 2 Department of Orthopaedics & Sports Medicine, University of South Florida, Morsani College of Medicine, 12901 Bruce B. Downs Blvd, MDC 8, Tampa, FL 33612, USA; 3 Department of Animal Science, University of California-Davis, College of Agricultural and Environmental Sciences, One Shields Ave, Davis, CA 95616, USA

## Abstract

**Introduction:**

Multipotent progenitor populations exist within the tendon proper and peritenon of the Achilles tendon. Progenitor populations derived from the tendon proper and peritenon are enriched with distinct cell types that are distinguished by expression of markers of tendon and vascular or pericyte origins, respectively. The objective of this study was to discern the unique tenogenic properties of tendon proper- and peritenon-derived progenitors within an *in vitro* model. We hypothesized that progenitors from each region contribute differently to tendon formation; thus, when incorporated into a regenerative model, progenitors from each region will respond uniquely. Moreover, we hypothesized that cell populations like progenitors were capable of stimulating tenogenic differentiation, so we generated conditioned media from these cell types to analyze their stimulatory potentials.

**Methods:**

Isolated progenitors were seeded within fibrinogen/thrombin gel-based constructs with or without supplementation with recombinant growth/differentiation factor-5 (GDF5). Early and late in culture, gene expression of differentiation markers and matrix assembly genes was analyzed. Tendon construct ultrastructure was also compared after 45 days. Moreover, conditioned media from tendon proper-derived progenitors, peritenon-derived progenitors, or tenocytes was applied to each of the three cell types to determine paracrine stimulatory effects of the factors secreted from each of the respective cell types.

**Results:**

The cell orientation, extracellular domain and fibril organization of constructs were comparable to embryonic tendon. The tendon proper-derived progenitors produced a more tendon-like construct than the peritenon-derived progenitors. Seeded tendon proper-derived progenitors expressed greater levels of tenogenic markers and matrix assembly genes, relative to peritenon-derived progenitors. However, GDF5 supplementation improved expression of matrix assembly genes in peritenon progenitors and structurally led to increased mean fibril diameters. It also was found that peritenon-derived progenitors secrete factor(s) stimulatory to tenocytes and tendon proper progenitors.

**Conclusions:**

Data demonstrate that, relative to peritenon-derived progenitors, tendon proper progenitors have greater potential for forming functional tendon-like tissue. Furthermore, factors secreted by peritenon-derived progenitors suggest a trophic role for this cell type as well. Thus, these findings highlight the synergistic potential of including these progenitor populations in restorative tendon engineering strategies.

## Introduction

Native repair of tendon following an injury such as a rupture or a tear is achieved through intrinsic and extrinsic mechanisms. When an injury occurs, leukocytes and fibroblasts migrate into the lesion early in repair [1]. Recently, it was determined that many of these fibroblasts originate from the paratenon and are thus an extrinsic source of cells involved in repair [2]. Moreover, cells from within the endotenon are thought to serve as an intrinsic source for repair, including tenocytes. Besides terminally differentiated cells such as leukocytes extrinsically and tenocytes intrinsically, multipotent progenitor populations also have been demonstrated to exist within the tendon proper and peritenon (paratenon and epitenon) of the tendon [3,4]. Progenitor populations derived from the tendon proper and peritenon of the Achilles tendon are enriched with distinct cell types that are distinguished by expression of markers of tendon and vascular/pericyte origins, respectively [4]. Specifically, isolated progenitors of the tendon proper express greater levels of scleraxis and tenomodulin while progenitors of the peritenon express greater levels of endomucin and prominin 1 [4]. Preliminary studies indicate that progenitor cells can be found within repair tissue of healing patellar tendon injuries [5]. Thus, stem/progenitor cells also may contribute to intrinsic and extrinsic tendon repair mechanisms.

Given the distinctions previously seen in the progenitor cell populations, we hypothesized in this study that tendon proper-derived stem/progenitors and peritenon-derived stem/progenitors possess differing tenogenic properties. Thus, we hypothesized that when incorporated into an *in vitro* regenerative model, progenitors from each region will respond uniquely and produce constructs with differing tenogenic properties. Tenogenic properties include: collagen-rich protein composition, features of the hierarchical tendon structure, as well as expression of known tenocyte markers [4,6-12]. To test these hypotheses, isolated peritenon- and tendon proper-derived progenitors were seeded within a contracting fibrinogen/thrombin gel-based tendon construct regenerative tissue model [4,13-18]. Growth differentiation factor 5 (GDF5) has been shown to influence tenocyte differentiation and potentially tendon development, as well as tendon homeostasis and tendon repair [9,19-24]. Thus, additionally, we hypothesized that differences in tenogenic properties will also be seen when each seeded progenitor population is treated with GDF5. Finally, because intrinsic and extrinsic mechanisms could work simultaneously within injured tendon via cell-cell crosstalk among cell types, conditioned media experiments were also performed to further define interactions among the two enriched progenitor cell populations and mature tenocytes, or more specifically the outcomes of paracrine signaling.

## Methods

### Animals

Ninety-six thirty-day-old (P30) male *ScxGFP* mice were used for twenty-four total progenitor and tenocyte isolations. Tendons also were dissected from embryonic *ScxGFP* mice (embryonic day (E)15 to E17) (n = 4) for controls in ultrastructural comparisons and from postnatal day P1 *ScxGFP* mice (n = 2) for controls in immuno-histochemical analyses of tendon constructs. *ScxGFP* mice contain the transgenic tendon promoter *Scx*-driven reporter *GFP*[25]. The University of South Florida’s Institutional Animal Care and Use Committee specifically approved this study’s protocols (‘USF R3901’).

### Progenitor cell isolation

Mice were euthanized and Achilles tendons with surrounding paratenon tissues were dissected using a sterile technique, and kept on ice in Dulbecco’s phosphate buffered saline (D-PBS, Life Technologies, Benicia, CA, USA) with antibiotics-antimycotics (100 U/ml penicillin, 100 μg/ml streptomycin, 250 ng/ml amphotericin B, Life Technologies). Each biological replicate represents eight Achilles tendons from four mice. Progenitor cells were isolated from the Achilles tendon proper as well as from the associated peritenon (paratenon and epitenon), utilizing a series of enzyme digestions as previously described [3,4,26]. Briefly, dissected tendons were first treated with 0.5% type I collagenase (CLS-1, Worthington, Lakewood, New Jersey, USA) and 0.25% trypsin (Life Technologies) in alpha minimum essential medium (alpha-MEM) for 10 minutes at 37°C. Then the surfaces of the tendons were scraped carefully with a rubber policeman to strip away the cells of the peritenon. Peritenon cells were collected in ice-cold alpha-MEM. The remaining tendon tissue was rinsed in Hank’s Balanced Salt Solution, cut into 1-mm^3^ pieces, and transferred into a solution of 3 mg/ml CLS and 4 mg/ml Dispase II (Roche, Basel, Switzerland). The digestion was undertaken at 90 oscillations per minute in a 37°C water bath for 20 minutes. Then digested tendon proper was transferred to culture media kept on ice, and fresh collagenase/dispase solution was transferred into the tube with the remaining tendon pieces for 10-minute incubations until all the tissue was digested, each time adding the digest to the media (alpha-MEM, 2 mM L-glutamine, antibiotics/antimycotics, 100 μm 2-mercaptoethanol, 20% fetal bovine serum (FBS)). From both digests (peritenon and tendon), cells were strained with a 70 μm cell strainer. Cells were collected by centrifugation for five minutes at 500 g, resuspended in media and counted using a hemocytometer with Trypan blue staining. Tendon and peritenon cells were plated in tissue culture flasks at 40 cells and 320 cells per cm^2^, respectively, so that adhering cells form segregated colonies within the flasks. Fourteen days into the culture, primary culture isolations of progenitor cell colonies were collected and used for generating tendon constructs or for the conditioned media experiment.

### Tenocyte isolation

Tenocytes were isolated from the tendon proper by removing the paratenon with enzyme digestion as described above. The remaining tendon tissue was rinsed in Hank’s Balanced Salt Solution, cut into 1-mm^3^ pieces, and transferred into a type I collagenase/Dispase II solution, also as described above. After digestion, cells were strained with a 70 μm cell strainer, collected by centrifugation for five minutes at 500 g, and resuspended in media. Tenocytes were plated in tissue culture flasks (50,000 cells per T75), and were grown in alpha-MEM with 2 mM L-glutamine, antibiotics-antimycotics, 10% FBS. After fourteen days into the culture, tenocytes also were used in the conditioned media experiment.

### Tendon constructs

**Figure 1:**
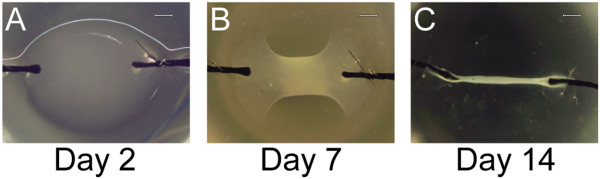
**Progenitor-seeded fibrin gels form tendon-like structures. (A)** Stem/progenitor cells are seeded within fibrinogen/thrombin; at Day 2, a distinct gel bridges the two silk sutures. **(B)** By Day 7, the fibrin within the structure has commenced contracting. **(C)** By Day 14, the construct has contracted into a long, narrow structure in which tension is applied uniaxially. Representative images are from tendon proper-derived stem/progenitor-cell-seeded constructs (Bar: 200 μm).

Tendon proper- and peritenon-derived progenitor cells were seeded into a regenerative tendon construct model as described previously [4,16]. Briefly, wells of six-well plates were coated with SYLGARD polymer (Dow Chemical, Midland, MI, USA). Within each well, two segments of size 0 silk were each pinned with a pair of minutiens insect pins (0.1 mm diameter, Fine Science Tools GmbH, Heidelberg, Germany) in each of the two suture segments positioned 10 mm apart. The contents of each well were sterilized by treatment with 100% ethanol, exposure to ultraviolet irradiation for 60 minutes and then rinsed in PBS. Within each well, 6.15 × 10^5^ tendon proper or peritenon progenitor cells in 400 μl media, 83 μl of 20 mg/mL fibrinogen, and 10 μl of 200 U/mL thrombin (Sigma, St Louis, MO, USA) were combined and quickly spread over the polymer surface between the two suture segments. Plates were incubated at 37°C in alpha-MEM with 10% FBS, 100 U/ml penicillin, 100 μg/mL streptomycin, 250 ng/ml amphotericin B, 2 mM L-glutamine and 200 μM ascorbic-2-phosphate, with or without GDF5 (100 ng/ml r-mGDF5, R&D Systems, Minneapolis, MN, USA) [4,23,27]. Three times per week the plates were scored to release the fibrin gel as it contracted as previously described [16], and the culture medium was changed. Over the first two weeks, gels contracted and tendon constructs formed (Figure 1). Cultures were maintained for up to 45 days.

### Conditioned media experiment

After the initial expansion, stem/progenitor cells or tenocytes were trypsinized from flasks and seeded in monolayer culture in 12-well plates (5.0 × 10^4^/well). Cells were maintained in their respective phenotype maintenance media: stem/progenitor (alpha-MEM, 2 mM L-glutamine, antibiotics/antimycotics, 100 μm 2-mercaptoethanol, 20% FBS) [3,4] or tenocytes (alpha-MEM, 2 mM L-glutamine, antibiotics/antimycotics, 0.15 mM ascorbate-2-phosphate, 10% FBS) [28] for 24 hours. Then, cells were rinsed with D-PBS and all cells received alpha-MEM, 1% FBS, L-glutamine, and antibiotics. After 18 hours, media were collected and grouped as tenocyte-conditioned (Tn), tendon-proper-stem/progenitor-conditioned (TP), and peritenon-stem/progenitor-conditioned (PERI). Then each cell type was treated with the conditioned media for 48 hours before cultures were terminated. Total RNA was isolated from the cell layer in each well.

### Electron microscopic examination of tenogenic differentiation

After 45 days in culture, tendon constructs were processed for transmission electron microscopy as described previously for tendons [4,29,30]. Biological replicates from each sample group (n = 3 to 8) were sectioned using a Leica Ultracut UCT ultramicrotome, and stained with 2% aqueous uranyl acetate and 1% phosphotungstic acid, pH 3.2. Microscopy was undertaken using a JEOL 1400 Transmission Electron Microscope. Images were digitally captured using a Gatan Orius widefield side mount CC Digital camera. Eight cross-sectional images from non-overlapping regions of the central portion of the construct taken at an instrument magnification of 60,000x were used for measuring diameters of fibrils formed within each biological replicate. Digital images were randomized, masked and transferred to a RM Biometrics-Bioquant Image Analysis System (Memphis, TN, USA) for analysis. Five to eight identical regions of interest (ROIs) per image were used on each image. All fibrils within the predetermined ROIs were measured until at least 100 fibrils were counted per image.

### Gene expression profiling

After 7 and 45 days in culture, total RNA was extracted from tendon proper- and peritenon-derived progenitor-seeded constructs by mechanically homogenizing tissues with a Tissue-Tearor (Biospec Products, Inc., Bartlesville, OK, USA) in QIAzol reagent (Qiagen, Valencia, CA, USA) and then using the QIAGEN RNeasy Plus Micro Kit [31]. Total RNA was isolated from cells in monolayer culture (pre-seeded cells compared to constructs or monolayer cultures for conditioned media studies) using the QIAGEN RNeasy Plus Micro Kit. For quantitative real-time polymerase chain reactions (RT-qPCR), total RNA (pre-seeded cells and tendon constructs, 250 ng; conditioned media monolayer culture, 200 ng) for each sample was reverse transcribed into cDNA using a High Capacity cDNA Reverse Transcription Kit (Applied Biosystems, ABI, Foster City, CA, USA). This cDNA was used as a template for RT-qPCR with Fast-SYBR Green Master Mix (ABI). Assays were performed using the StepONEPlus Real-Time PCR System (ABI). Primers were designed using Primer Express software (ABI) for the following genes: actin, beta (*Actb*); scleraxis (*Scx*); tenomodulin (*Tnmd*); endomucin (*Emcn*); biglycan (*Bgn*); decorin (*Dcn*); collagen, type I, alpha 1 (*Col1a1*); collagen, type III, alpha 1 (*Col3a1*); collagen, type V, alpha 1 (*Col5a1*); collagen, type XI, alpha 1 (*Col11a1*); collagen, type XII, alpha 1 (*Col12a1*); and collagen, type XIV, alpha 1 *(Col14a1)*. Primer sequences can be found in Table 1. Two technical replicates were performed for each biological replicate (n = 5 to 8 per group for tendon constructs; n = 4 to 8 for the conditioned media experiment). The results were adjusted for efficiency as measured by LinRegPCR using the default fit option that measures the slope of a line containing four to six data points and the highest R^2^ correlation value [32,33]. RT-qPCR data were normalized relative to the endogenous control gene, *Actb*.

**Table 1:** Sequence information for primers used in RT-qPCR

**Gene symbol**	**Forward primer sequence**	**Reverse primer sequence**
*Actb*	5-AGATGACCCAGATCATGTTTGAGA-3	5-CACAGCCTGGATGGCTACGT-3
*Bgn*	5-CTACGCCCTGGTCTTGGTAA-3	5-ACTTTGCGGATACGGTTGTC-3
*Col1a1*	5-TTCTCCTGGCAAAGACGGACTCAA-3	5-AGGAAGCTGAAGTCATAACCGCCA-3
*Col3a1*	5-CACGCAAGGCAATGAGACTA-3	5-TGGGGTTTCAGAGAGTTTGG-3
*Col5a1*	5-AAGCGTGGGAAACTGCTCTCCTAT-3	5-AGCAGTTGTAGGTGACGTTCTGGT-3
*Col11a1*	5-CTGGTCATCCTGGGAAAGAA-3	5-AGCCCTTGAGACCTCTGACA-3
*Col12a1*	5-TGACTACGGTGCAGATGAGC-3	5-AAGCGACGCAGAGAAAACAT-3
*Col14a1*	5-ACCTGTGAGTGTCCCTGGTC-3	5-AGGCCAGTCAGAGCATCACT-3
*Dcn*	5-TGAGCTTCAACAGCATCACC-3	5-AAGTCATTTTGCCCAACTGC-3
*Emcn*	5-CCAACAGTCTCTGCCACAGTGA-3	5-ACAGAGGCTTTTGTTGTGGAAGTT-3
*Scx*	5-AAGTTGAGCAAAGACCGTGACA-3	5-TGTGGACCCTCCTCCTTCTAAC-3
*Tnmd*	5-CGCCACACCAGACAAGCA-3	5-CCAGCATTGGGTCAAATTCA-3

### Immuno-histochemical analysis of tendon constructs

Tendon construct protein content was analyzed alongside of one day old (P1) ScxGFP mouse Achilles tendons using an immuno-histochemical technique previously described [34,35]. Briefly, dissected P1 Achilles tendons and 7-day tendon constructs were fixed in 4% paraformaldehyde in phosphate-buffered saline (PBS), pH 7.2, for two to four hours at 4°C, then stored in an increasing gradient of sucrose and embedded in Optimum Cutting Temperature (OCT) compound (Tissue-Tek^®^, Sakura Finetek USA, Torrance, CA, USA). Five-micron cryo-sections were then generated and placed on Superfrost Plus slides (Fisher). Slides were rinsed twice in PBS, quenched with 1% glycine in PBS and blocked with 2% bovine serum albumin (BSA) in PBS at room temperature for one hour. Primary antibodies were applied with 1% BSA in PBS overnight at 4°C and then rinsed twice with PBS containing 0.2% Tween-20. Secondary antibodies were applied with 1% BSA in PBS at room temperature for one hour and then slides were rinsed with PBS containing 0.2% Tween-20 and PBS. Coverslips were applied to slides after application of Vectashield^®^ mounting solution with 4′,6-diamidino-2-phenylindole (DAPI) (1:1 H-1000 to H-1200, Vector Laboratories, Inc., Burlingame, CA, USA) used as a nuclear marker. Negative control samples were incubated identically, except without primary antibody. The primary antibodies that were used included: 10 μg/ml rabbit anti-mouse collagen I (AB765P, EMD Millipore, Corp., Billerica, MA, USA), 1:200 rabbit anti-mouse collagen III (LB-1393, Cosmo Bio USA, Carlsbad, CA, USA), and 1:100 rabbit anti-mouse collagen V (Myriad Genetic Laboratories, Inc., Salt Lake City, UT 84108, USA) [36]. The secondary antibody was an Alexa Fluor 568-conjugated goat anti-rabbit immunoglobulin G (IgG) (Molecular Probes, Eugene, OR, USA) used at 1:200. Images were captured using a DM5500 upright microscope system (Leica) with conditions and integration times set to facilitate comparisons between samples.

### Statistical analyses

These studies utilized both general distribution descriptions as well as tests of statistical significance. Features of the fibril diameter distributions for each construct group included mean fibril diameters, standard deviations, median diameter, and first and third quartile values. Statistical analyses of fibril diameter distributions were performed using the unpaired two-tailed *t*-test comparing mean fibril diameters of each image analyzed for each group (E15-17, TP, TP + GDF5, PERI, PERI + GDF5). RT-qPCR results for progenitor-seeded construct profiles and for expression profiling results from the conditioned media study were analyzed using non-parametric Mann–Whitney-Wilcoxon tests on unpaired samples.

## Results

### Ultrastructural analysis of progenitor-derived matrix assembly

**Figure 2:**
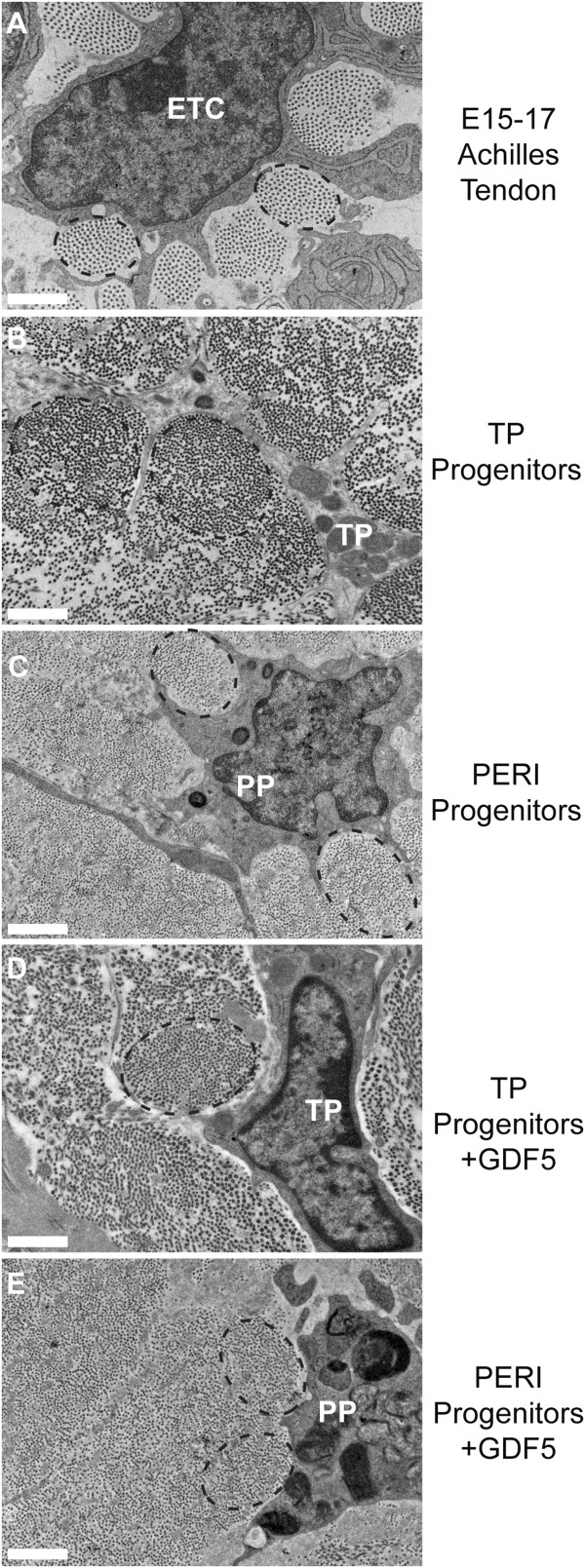
**Progenitor-derived matrix assembly is ultrastructurally similar to that of embryonic tendon.** Ultrastructure was examined by cross-sectional images. In both the E15 to E17 Achilles tendon **(A)** and the progenitor constructs **(B-E)**, collagen fibril synthesis and fiber assembly by cell processes are evident with fibers labeled with black dashed-line ovals **(A-E)**. Panels: Embryonic Achilles tendon **(A)**, tendon proper (TP)-derived progenitor-seeded construct **(B)**, peritenon (PERI)-derived progenitor-seeded construct **(C)**,TP-derived progenitor-seeded construct supplemented with GDF5 **(D)**, peritenon (PERI)-derived progenitor-seeded construct supplemented with GDF5 **(E)**. ETC: embryonic tendon cell; TP: tendon proper-derived progenitor; PP: peritenon-derived progenitor (Bar: 1 μm). E, embryonic day; GDF5, growth differentiation factor 5.

This series of experiments tested the hypothesis that the tendon proper and peritenon progenitors have unique tenogenic properties when incorporated into the *in vitro* regenerative model. The structures of the engineered tissues and the developing tendons were analyzed using transmission electron microscopy. The progenitor-seeded constructs generated tendon-like tissues with a structure similar to that of embryonic tendon. That is, after 45 days in culture, the ultrastructure analysis of cross-sectional images from the mid-regions of the constructs shows that cells seeded within the constructs have processes projecting into the matrix compartmentalizing the extracellular matrix and organizing collections of fibrils into fibers, all of which is comparable to that observed in embryonic (E15 to E17) Achilles tendon (Figure 2A-E). Qualitatively, this is seen for constructs seeded with tendon proper- or peritenon-derived progenitors when cultured with or without supplementation of GDF5.

**Figure 3:**
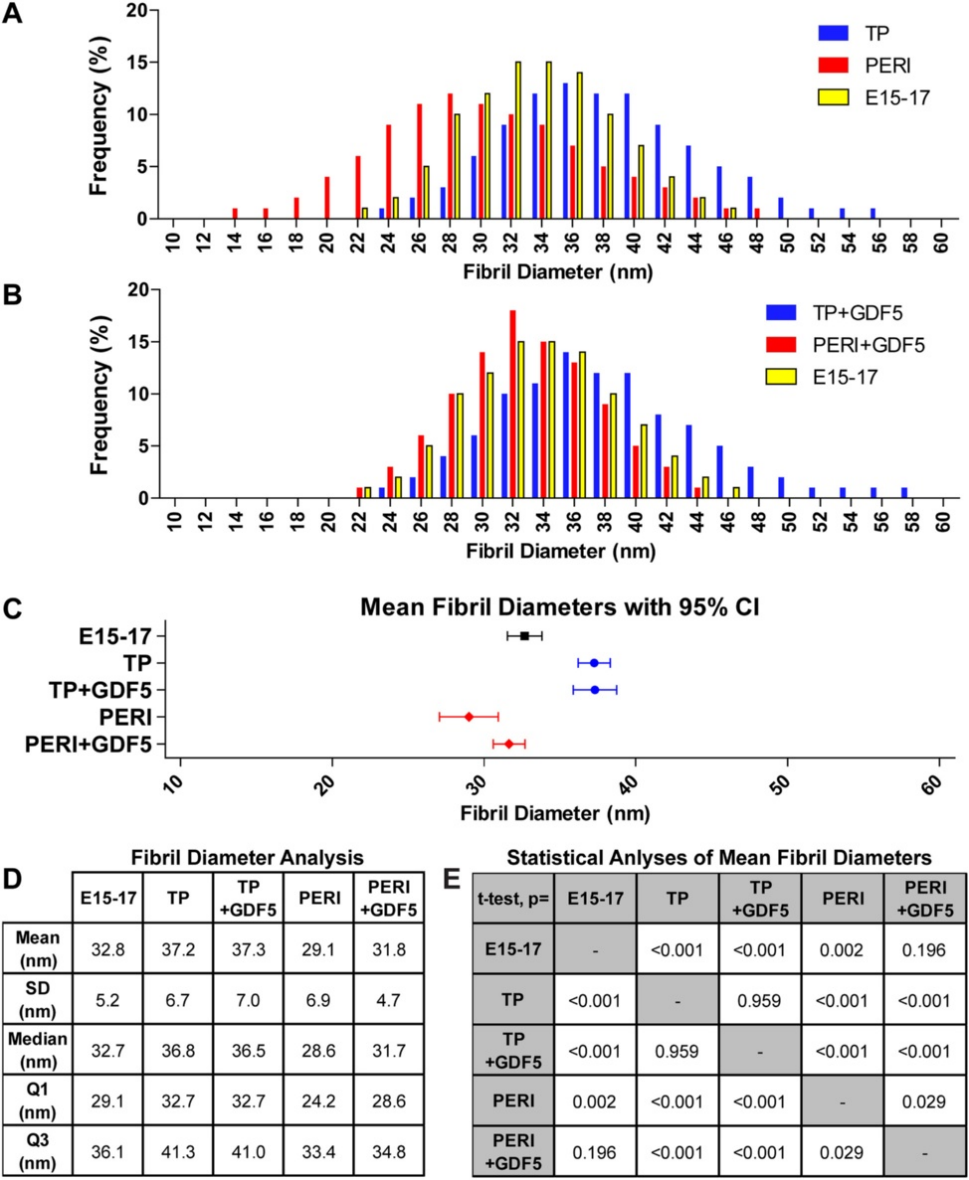
**Mean fibril diameters for constructs were similar to embryonic tendon, and supplemented GDF5 improved peritenon progenitor-derived construct mean fibril diameters.** Fibril diameters of tendon proper progenitor-seeded (TP) constructs were greater than those of the peritenon-derived progenitor (PERI) constructs as well as those of embryonic Achilles tendon (E15 to E17) **(A)**. Supplementation with GDF5 increased fibril diameters within PERI constructs **(B)** but not TP constructs. For each group, the mean fibril diameters with 95% confidence interval (CI) are compared **(C)**, and fibril diameter analyses are also given for each group **(D)** – mean fibril diameters ± standard deviation, median, first and third quartiles. Significant differences are demonstrated via *t*-test statistical analyses of fibrils analyzed in each group’s images **(E)**. Replicates (n) represent each image analyzed per group: E15 to E17, n = 32; TP, n = 64; TP + GDF5, n = 32; PERI, n = 32; PERI + GDF5, n = 24. E, embryonic day; GDF5, growth differentiation factor 5.

An analysis of fibril diameters generated within the constructs revealed that overall both progenitor populations assembled fibrils with embryonic tendon-like features, that is, unimodal diameter distributions with homogeneous small diameters. Fibrils assembled by the tendon proper-derived progenitors demonstrated a broader diameter distribution shifted to larger diameters than in the embryonic tendon with larger mean diameters (37.2 ± 6.7 nm versus 32.8 ± 4.7 nm, mean ± sd, *P* <*0*.001) (Figure 3A,D,E). The peritenon-derived progenitor distribution was shifted to smaller diameters (29.1 ± 6.9 nm, *P* = 0.002) (Figure 3A, D,E). However, when GDF5 is supplemented, peritenon-derived progenitors produce fibrils with a mean diameter distribution (31.8 ± 4.7 nm) comparable to that of embryonic tendon (*P* = 0.196; Figure 3B,D,E) and no change in the fibril diameters (37.8 ± 4.7 nm) is noted for tendon proper-derived progenitors (*P* = 0.959; Figure 3B,D,E).

### Gene expression analyses of progenitors in tendon-like structures

**Figure 4:**
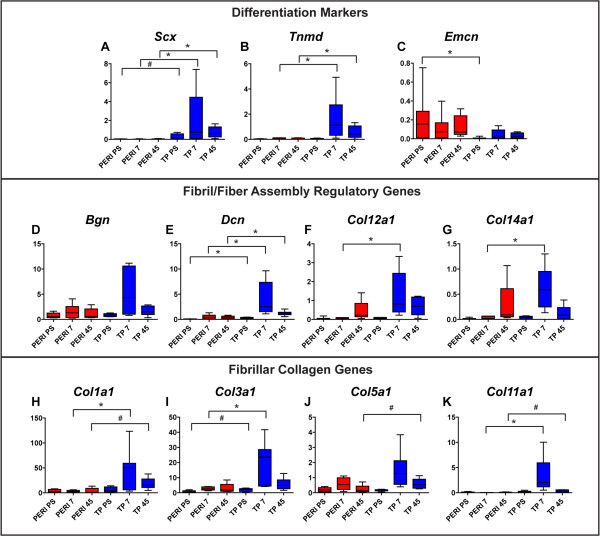
**Expression of tendon differentiation markers and matrix assembly genes is generally greater in tendon proper-derived progenitors.** RT-qPCR analyses demonstrated greater expression of the tendon markers *Scx***(A)** and *Tnmd***(B)** in tendon proper (TP)-derived progenitors within their respective constructs. However, expression of the vascular marker *Emcn***(C)** was greater in peritenon-derived (PERI) progenitors. Expression of *Bgn***(D)** remains fairly constant across cell sources, while expression for *Dcn***(E)** is greater in TP-derived progenitor-seeded tendon constructs. Expression of FACIT collagens *Col12a1***(F)** and *Col14a1***(G)** is greater early in culture, particularly for TP-derived progenitors. Gene expression for fibril-forming collagens *Col1a1***(H)**, *Col3a1***(I)**, and *Col5a1***(J)** is greater in TP-derived progenitors. Expression of *Col11a1***(K)** is greatest early in TP-derived progenitors. (Biological replicates, n = 5 to 8; Mann–Whitney-Wilcoxon test – *P* <0.01, *; *P* <0.05, #; fold changes and statistical significance further reported in Table 2).

To test our hypothesis that tendon proper- and peritenon-derived progenitors have different tenogenic capacities within the *in vitro* regenerative model, we analyzed expression of differentiation markers and matrix assembly genes early (Day 7) and late (Day 45) in construct formation (Table 2). Expression of tendon differentiation markers *Scx* and *Tnmd* was shown to be different in pre-seeded tendon proper-derived and peritenon-derived progenitors [4]; in this study, expression of *Scx* is greater in tendon proper-derived progenitors and trends higher for *Tnmd* (Figure 4A,B; Table 2). Expression of *Scx* and *Tnmd* was significantly greater in tendon proper-derived progenitors seeded in constructs (Figure 4A,B; TP versus PERI: 30- to 90-fold and 13- to 21-fold, respectively), while expression of vascular marker *Emcn* had a greater expression level in pre-seeded peritenon-derived progenitors and a greater trend of expression in peritenon-derived progenitors (Figure 4C; Table 2). Transcript levels for *Bgn* were relatively equivalent across cultures (group) and stage (days) (Figure 4D), but levels for *Dcn* were significantly greater in tendon proper-derived progenitors (Figure 4E). FACIT collagen genes *Col12a1* and *Col14a1* were significantly greater in tendon proper-derived progenitors at the early time point (Figure 4F,G). Expression of fibrillar collagen genes *Col1a1*, *Col3a1*, *Col5a1*, and *Col11a1* were generally greater in tendon proper-derived progenitors seeded in constructs both early and late in culture (Figure 4H-K).

**Table 2:** Mean expression fold differences of TP and PERI constructs

**Gene**	**TP PS/PERI PS**	**TP 7/PERI 7**	**TP 45/PERI 45**
***Scx***	**10.55**^**a**^	**94.49**^**b**^	**29.58**^**b**^
***Tnmd***	2.07	**20.90**^**b**^	**12.90**^**b**^
***Emcn***	**0.03**^**b**^	0.80	0.23
***Bgn***	1.13	3.66	1.54
***Dcn***	**4.92**^**b**^	**6.18**^**b**^	**4.44**^**b**^
***Col1a1***	1.76	**5.58**^**b**^	**4.75**^**a**^
***Col3a1***	**2.51**^**a**^	**3.30**^**b**^	2.63
***Col5a1***	0.82	2.57	**2.26**^**a**^
***Col11a1***	2.71	**97.40**^**b**^	**5.40**^**a**^
***Col12a1***	0.87	**3.61**^**b**^	1.62
***Col14a1***	2.09	**13.42**^**b**^	0.44

Expression fold differences represent the ratio of means of (Target Gene/*Actb*)_Group A_/(Target Gene/*Actb*)_Group B_. PERI: peritenon-derived progenitors; TP: tendon proper-derived progenitors. Days in culture: pre-seeded (PS), 7 days in construct (7), 45 days in construct (45). Statistical significance of fold changes are in **bold** (^a^*P* <0.05; ^b^*P* <0.01); two-tailed unpaired Mann–Whitney-Wilcoxon tests were performed. Graphical representations of the data are found in Figure 4.

**Figure 5:**
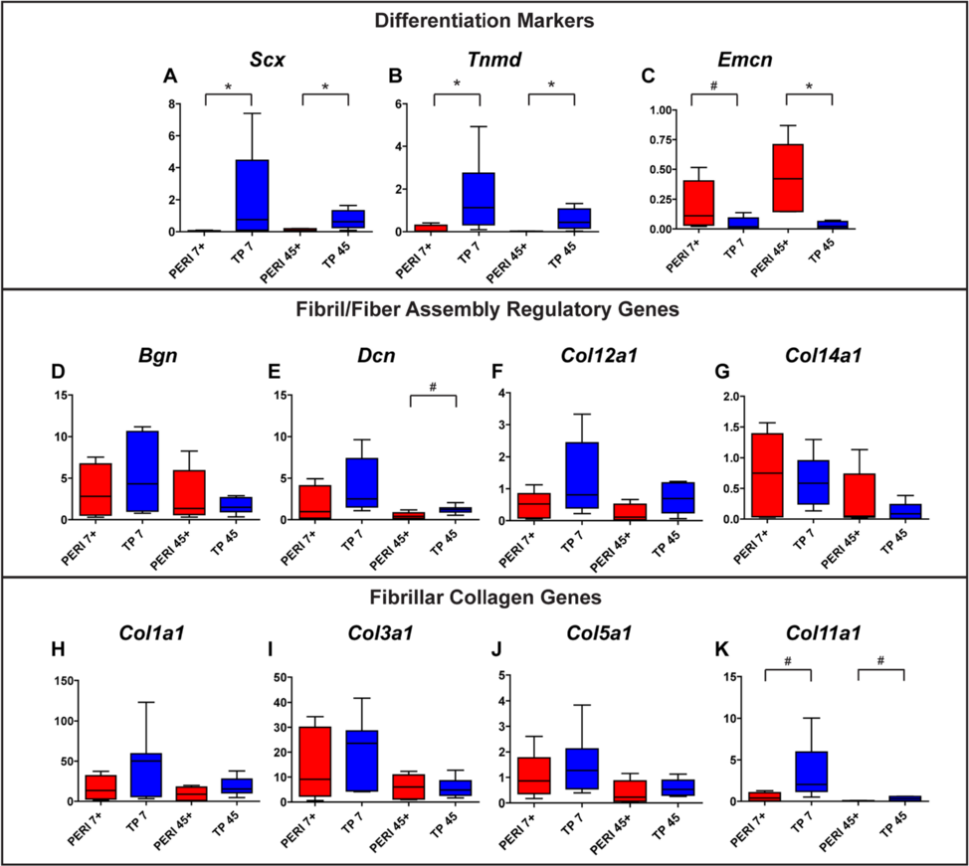
**GDF5 supplementation causes improved expression matrix assembly genes in peritenon progenitors seeded in constructs.** After r-mGDF5 supplementation, expression levels for the tendon markers *Scx***(A)** and *Tnmd***(B)** and the vascular marker *Emcn***(C)** still differed. GDF supplementation led to improved expression of matrix assembly genes for peritenon progenitors seeded in tendon constructs - comparing PERI + versus TP at Day 7 (early) and Day 45 (late). Bolstered expression is noted for SLRPs *Bgn***(D)** and early for *Dcn***(E)**, for FACIT collagens *Col12a1***(F)** and *Col14a1***(G)**, and for fibril-forming collagens *Col1a1***(H)**, *Col3a1***(I)** and *Col5a1***(J)**. Expression of *Col11a1***(K)** is still greater for tendon proper-derived progenitors. (Biological replicates, n = 5 to 8; Mann–Whitney-Wilcoxon test – *P* <0.01, *; *P* <0.05, #; fold changes and statistical significance further reported in Table 3 and expanded results are reported in Additional file 1: Figure S1). GDF5, growth differentiation factor 5; PERI, peritenon-derived progenitors; SLRP, small leucine-rich proteoglycans; TP, tendon proper-derived progenitors.

When the culture was supplemented with recombinant mouse GDF5, expression for matrix assembly genes in seeded peritenon progenitors increased to levels seen with the seeded tendon proper-derived progenitors (Table 3). However, after GDF5 supplementation, expression levels for the tendon markers *Scx* (Figure 5A) and *Tnmd* (Figure 5B) were unchanged when comparing GDF5-supplemented peritenon progenitor expression (*PERI+*) to that of tendon proper-derived progenitors not supplemented with GDF5 (*TP*). Expression of vascular marker *Emcn* (Figure 5C) also still differed between PERI + and TP progenitors. GDF5 supplementation did result in improved expression of matrix assembly genes for peritenon progenitors seeded in tendon constructs comparing *PERI +* progenitors and *TP* progenitors. Bolstered expression is noted for the small leucine-rich proteoglycans (SLRPs) *Bgn* early (Figure 5D) and *Dcn* (Figure 5E). Expression of FACIT collagens *Col12a1* (Figure 5F) and *Col14a1* (Figure 5G) as well as fibril-forming collagens *Col1a1* (Figure 5H), *Col3a1* (Figure 5I), and *Col5a1* (Figure 5J) for GDF5-supplemented *PERI +* progenitors also became comparable to TP progenitors, yet expression of *Col11a1* (Figure 5K) was still greater for TP progenitors. Addition of GDF5 did not lead to significant expression changes among TP progenitors for the tendon markers and matrix assembly genes analyzed, except for *Bgn* at 45 days; Additional file 1: Figure S1 exhibits expression results for constructs generated by cell type, GDF5 supplementation and culture time.

**Table 3:** Mean expression fold differences of PERI constructs given GDF5 versus TP constructs

**Gene**	**TP 7/PERI 7 + GDF5**	**TP 45/PERI 45 + GDF5**
***Scx***	**64.16**^**b**^	**7.75**^**b**^
***Tnmd***	**13.41**^**b**^	**37.85**^**b**^
***Emcn***	**0.21**^**a**^	**0.07**^**b**^
***Bgn***	1.67	0.59
***Dcn***	1.69	**3.26**^**a**^
***Col1a1***	2.81	1.96
***Col3a1***	1.38	1.30
***Col5a1***	1.40	1.29
***Col11a1***	**6.46**^**a**^	**6.52**^**a**^
***Col12a1***	2.43	2.95
***Col14a1***	0.84	0.39

Expression Fold Differences represent the ratio of means of (Target Gene/*Actb*)_Group A_/(Target Gene/*Actb*)_Group B_. PERI: peritenon-derived progenitors; TP: tendon proper-derived progenitors. Days in culture: 7 days in construct (7), 45 days in construct (45); +: with GDF5 supplementation. Statistical significance of fold changes are in **bold** (^a^*P* <0.05; ^b^*P* <0.01); two-tailed unpaired Mann–Whitney-Wilcoxon tests were performed. Graphical representations of the data are found in Figure 5.

### Immuno-histochemial analysis of protein content

**Figure 6:**
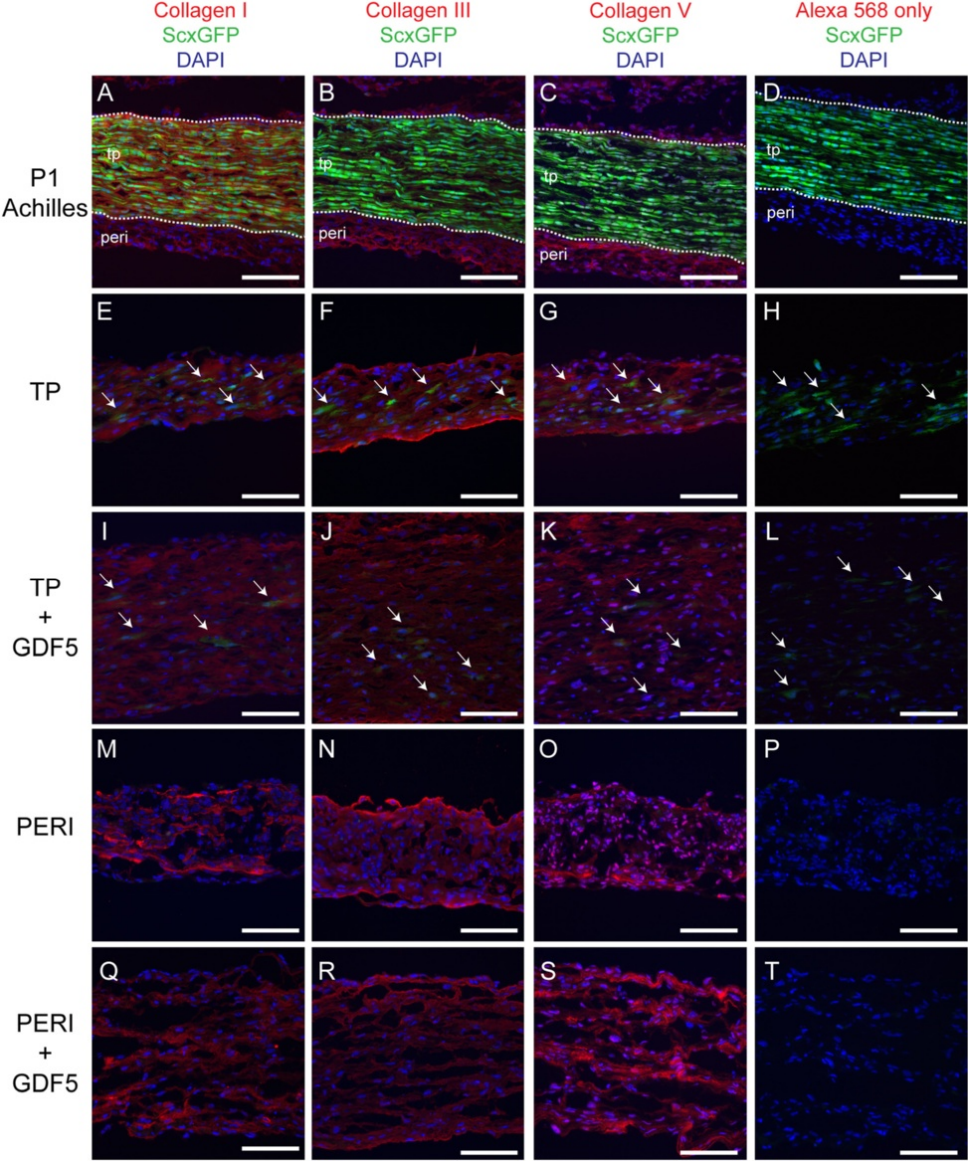
**Fibrillar collagens were present in the tendon constructs.** Staining for collagens I, III and V was analyzed in P1 Achilles tendon and progenitor-derived tendon constructs at Day 7. Because the tissue and cells originate from ScxGFP transgenic mice, cells with an active *Scx* promoter contain green fluorescent protein (GFP). In the P1 Achilles tendon, GFP allows for delineation (white dashed line) of peritenon (peri) and tendon proper (tp) **(A-D)**. Active GFP was also detected for TP-derived progenitors in constructs (white arrows, **E-L**). As with the P1 Achilles tendon, TP, TP + GDF5, PERI, and PERI + GDF5 constructs were positive for staining for collagen I **(A, E, I, M, Q)**, collagen III **(B, F, J, N, R)** and collagen V **(C, G, K, O, S)**. Negative controls represent staining with secondary antibody only **(D, H, L, P, T)** (Bar: 100 μm).

To analyze levels of differentiation markers and matrix assembly proteins within the constructs, expression of scleraxis, collagen I, collagen III, and collagen V in early (Day 7) constructs was evaluated using immuno-histochemical analyses. Scleraxis expression was visualized via the *Scx* promoter-driven green fluorescent protein (GFP) reporter present within cells derived from the *ScxGFP* mouse line [25]. GFP was detected in all tenocytes of P1 Achilles tendon yet not in cells of the surrounding peritenon (Figure 6A-D) Moreover, fewer cells expressed GFP in TP progenitor-derived constructs (Figure 6E-L); however, GFP was virtually absent in peritenon progenitor-derived onstructs (Figure 6M-T), even when supplemented with GDF5. Expression of collagen I was detectable in Achilles tendon (Figure 6A) as well as the tendon constructs (Figure 6E,I,M,Q). Collagen III was detectable in Achilles tendon (Figure 6B) and the tendon constructs (Figure 6F,J,N,R). Likewise, collagen V was present in the P1 Achilles tendon (Figure 6C) and the tendon constructs (Figure 6G,K,O,S). Thus, expression of fibrillar collagens is confirmed in the tendon constructs, and differential expression of the tendon marker scleraxis among progenitor-derived constructs is corroborated by *Scx* promoter-driven GFP.

### Gene expression analyses from conditioned media experiment

**Figure 7:**
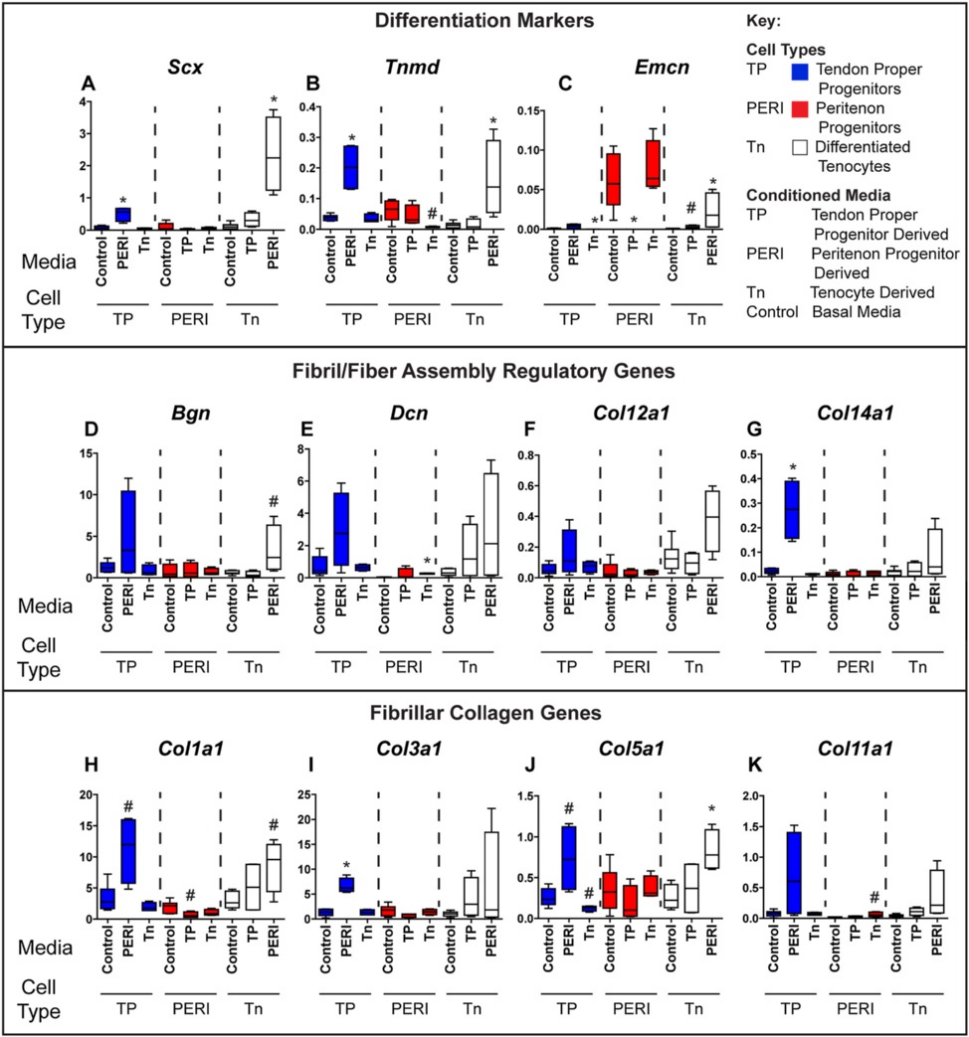
**Peritenon-derived progenitor conditioned media stimulates expression of tendon differentiation markers and matrix assembly genes.** Treatment of tendon proper-derived progenitors and tenocytes with peritenon-derived progenitor conditioned media (PERI) resulted in increased expression of the tendon markers *Scx***(A)** and *Tnmd***(B)**, as well as vascular marker *Emcn***(C)**. When tendon proper-derived progenitors and tenocytes were treated with PERI conditioned media, expression of matrix assembly genes increased as a trend for *Bgn***(D)**, *Dcn***(E)**, *Col12a1***(F)** and *Col11a1***(K)**; and significantly for *Col14a1***(G)**, *Col1a1***(H)**, *Col3a1***(I)** and *Col5a1***(J)**. Peritenon-derived progenitors were not stimulated by tendon proper-derived progenitor or tenocyte conditioned media. (Biological replicates, n = 4 to 8; Mann–Whitney-Wilcoxon test relative to control media for each cell type for each gene assayed – *P* <0.01, *; *P* <0.05, #).

During repair, several cell types present in the lesion offer the opportunity for crosstalk to occur among cells, thus affecting re-establishment of tendon form and function. To test the hypotheses that different progenitor populations present in healing tissue are capable of cross talk in the regenerative response, an *in vitro* monolayer culture model was utilized. Specifically, a conditioned media experiment was done to analyze interactions via paracrine stimulatory products. Gene expression analyses were used to discern the effects of the cell type-specific secretory products of peritenon-derived progenitors, tendon proper-derived progenitors, and tenocytes, resulting in nine cell type and media combinations - cell type (conditioned media): TP (control), TP (PERI), TP (Tn), PERI (control), PERI (TP), PERI (Tn), Tn (control), Tn (TP), Tn (PERI). Treatment of tendon proper-derived progenitors and tenocytes with peritenon-derived progenitor conditioned media resulted in increased expression of the tendon markers *Scx* (Figure 7A) and *Tnmd* (Figure 7B), as well as vascular marker *Emcn* (Figure 7C). When tendon proper-derived progenitors and tenocytes were treated with peritenon-derived progenitor conditioned media (PERI), expression of matrix assembly genes increased as a trend for *Bgn* (Figure 7D), *Dcn* (Figure 7E) and *Col12a1* (Figure 7F). Significant increases in expression were demonstrated for *Col14a1* (Figure 7G), *Col1a1* (Figure 7H), *Col3a1* (Figure 7I), *Col5a1* (Figure 7J) and *Col11a1* (Figure 7K) in tendon proper-derived progenitors and tenocytes when treated with peritenon-derived progenitor conditioned media. However, peritenon-derived progenitors were not stimulated by tendon proper-derived progenitor or tenocyte conditioned media.

## Discussion

Intrinsic and extrinsic cells, including stem/progenitors, have been described as plausible participants in the repair of tendon tears and ruptures [1,2]. In this study, we demonstrated that stem/progenitor populations of the tendon proper and peritenon of the Achilles tendon are capable of forming collagen fibril-rich tendon-like structures *in vitro* that are comparable to embryonic tendons. However, we found that there are several differences in each progenitor population’s ability to regenerate tendon. These *in vitro* differences provide insight into how these two progenitor populations may behave during native tendon repair or in tendon formation in tissue engineering.

While both progenitor populations produced tendon-like structures when seeded in fibrin gel constructs, as demonstrated in this study and previously [4], the tendon proper-derived progenitors demonstrated greater levels of tenogenic differentiation by expression of the tendon markers *Scx* and *Tnmd*. Even after supplementation with the tenogenic modulator GDF5, expression differences for these tenogenic marker genes still remained unchanged, although comparable levels of matrix assembly gene expression were noted. Expression differences of *Scx* and *Tnmd* in these progenitor populations signify the need for better understanding of the stem/progenitor cells involved in tendon repair as well as the markers that may define the origins and, thus, variable differentiation potentials of intrinsic and extrinsic cell populations [37,38]. Since cells from the peritenon enter into lesions early, these cells are proposed to transdifferentiate into tenocytes [2]. In the tendon regeneration model, peritenon-derived progenitors are not differentiating into tenocytes in a manner comparable to progenitors from within the tendon, as measured by *Scx* and *Tnmd* expression and *ScxGFP* transgenic reporter. Thus, from the *in vitro* results, it could be stated that not every cell that expresses matrix assembly genes has transdifferentiated into a tenocyte, but instead into another collagen-producing cell.

In the regeneration model, ultrastructural similarities were seen between tendon-like structures developed from peritenon- and tendon proper-derived progenitors. That is, progenitors seeded within the constructs had processes projecting into the matrix that gathered collections of fibrils for further assembly into fibers as in developing tendons. Also, likewise, these fibrils were aligned longitudinally, along the tendon axis. However, diameter distributions varied significantly between structures produced by tendon proper-derived progenitors and peritenon-derived progenitors. Differences in fibril diameters could be the result in differential expression of regulatory molecules, such as SLRPs and FACIT collagens, as well as fibril nucleating *Col5a1* and *Col11a1*[34]. The addition of GDF5 did stimulate matrix assembly gene expression in peritenon progenitors, subsequently contributing to the corresponding shift in fibril diameter distributions toward larger diameters. If this is the case, then this would suggest that GDF5 might also stimulate matrix assembly gene expression in peritenon progenitors through another tenogenic transcription factor, such as *Mohawk*, or the early growth response transcription factors *Egr1* and *Egr2*[14,27,39]. Certainly, transcription factors besides *Scx* are active in stimulating transcription of type I procollagen genes and other matrix assembly genes, even in the tendon [40,41]. Further studies are required to determine each transcription factor’s involvement in activation of intrinsic and extrinsic cells in repair.

In examining gene expression of tendon markers and matrix assembly early and late in fibrin gel-based construct cultures, gene expression waned to various extents from Day 7 to Day 45, particularly for TP constructs. However, the decrease in expression was not statistically significant, except for *Col11a1* and *Col14a1*, although decreases occur in expression for these two genes in developing tendons [34,42,43]. In fact, expression for many matrix assembly genes wanes over time as tendon development ensues, maturation proceeds and homeostasis occurs [6,9,34,42,44,45]. Whether or not this decrease in expression represents the construct reaching a homeostatic state is still something to be determined. This suggests that other stimuli might be necessary to further stimulate the constructs as they form tendon-like structures.

In the debate over the roles of intrinsic and extrinsic cell sources in repair, it is believed that extrinsic cells secrete stimulatory factors during repair [1,46]. Here we demonstrated that peritenon-derived progenitors secrete a factor or factors that bolster expression of tenogenic differentiation markers and matrix assembly genes in tendon proper-derived progenitors and mature tenocytes, thus suggesting another role for peritenon-derived progenitors besides synthesis of provisional matrix during tendon repair. This stimulatory effect also highlights the necessity for considering the interactions of all intrinsic and extrinsic cells involved in tendon repair, besides tendon niche components, breakdown products, as well as inflammatory mediators. In a recent study, the stimulatory effect of tendon cells has also been demonstrated to promote tenogenic differentiation in amniotic epithelial cells in co-culture [47]. Thus, cell-cell signaling could be invaluable for cuing tenogenic differentiation in native repair; likewise, manipulation of this signaling could lead to improvements in regenerative tissue engineering strategies.

This study did have a few limitations. Only one concentration of GDF5 was used in these studies. This concentration was selected because it had been effective for other mesenchymal stromal cell-based studies [23,27,48,49]. Furthermore, while this study focused on GDF5, several other growth differentiation factors have been implicated in tendon development and could also merit further investigation within the model [50-54]. Moreover, further analyses of morphological structure, particularly dynamic tracking techniques, would be useful for understanding cell behavior within the developing constructs as cells undergo tenogenic differentiation and initiate tendon formation [55-57].

It should be noted that prior to the conditioned media study, cells were expanded in media for a two week period and then cells were trypsinized and then re-seeded, which is essentially the first passage. Progenitors were expanded in progenitor selective media [3,4]. Tenocytes were expanded in media with 10% FBS; supplementation of media with 10% FBS has been demonstrated to be acceptable for tenocyte expansion [11,58], although phenotypic drift could become a concern after long periods of time or multiple passages [28]. Phenotypic drift related to long-term, multiple passage culture is also a concern for progenitor cells [59].

Lastly, the progenitors in this study were examined via *in vitro* models. The tendon-forming capabilities of progenitors were analyzed in the fibrin gel model, and enriched progenitor populations – tendon proper versus peritenon – were examined individually, although in native repair intrinsic and extrinsic sources may interact to influence repair, as was demonstrated by the conditioned media study. The purpose of the conditioned media study was to offer evidence of cell-cell interactions via paracrine effects to highlight the importance of considering more than one cell type within the healing niche. Future studies are planned to discern how this synergistic interaction can be influenced to regenerate tendon-specific structure and function in engineered constructs via co-culture as well as *in situ* during repair via cell tracking within healing tendon tissue.

## Conclusions

We demonstrated that progenitors from the tendon proper and the peritenon are capable of forming collagen-rich structures. While TPs are more suited for regeneration of tendon structure, the addition of GDF5 stimulated matrix assembly for PERIs. Moreover, PERIs secreted tendon-promoting factors that bolster expression of tendon markers in TPs and tenocytes. These findings suggest that progenitors in and around the Achilles tendon possess unique tenogenic differentiation characteristics. Distinctions found in tenogenic differentiation and matrix assembly potentials between these progenitor pools provide insight into possible individual and interactive roles of multiple intrinsic and extrinsic cell populations during tendon repair.

## Abbreviations

BSA: bovine serum albumin; CLS-1: type 1 collagenase; D-PBS: Dulbecco’s phosphate-buffered saline; FACIT: fibril associated collagens with interrupted triple helices; FBS: fetal bovine serum; GDF5: growth/differentiation factor-5; PERI: peritenon-derived progenitor or peritenon progenitor conditioned; ROI: region of interest; SLRP: small leucine-rich proteoglycan; Tn: tenocyte-derived or tenocyte-conditioned; TP: tendon proper-derived progenitor or tendon proper progenitor conditioned; alpha MEM: alpha minimum essential medium.

## Competing interests

The authors declare that they have no competing interests.

## Authors’ contributions

MJM conceived the hypotheses of the study. MJM and DEB participated in the design of the study. MJM carried out cell culture, gene expression and immuno-histochemistry studies. MJM and SMA performed ultrastructural analyses. MJM performed statistical analyses. All authors contributed to writing the manuscript. All authors read and approved the final manuscript.

## Supplementary Material

Additional file 1: Figure S1 GDF5 supplementation causes improved expression matrix assembly genes in peritenon progenitors seeded in constructs: an expanded view. After r-mGDF5 supplementation, expression levels for tendon markers *Scx***(A)** and *Tnmd***(B)** and vascular marker *Emcn***(C)** still differed. GDF5 supplementation led to improved expression of matrix assembly genes for peritenon progenitors seeded in tendon constructs -comparing PERI + vs. TP: Day 7 (early), red boxes; Day 45 (late), blue boxes. Bolstered expression is noted for SLRPs *Bgn***(D)** and early for *Dcn***(E)**, for FACIT collagens *Col12a1***(F)** and *Col14a1***(G)**, and for fibril-forming collagens *Col1a1***(H)**, *Col3a1***(I)**, and *Col5a1***(J)**. Expression of *Col11a1***(K)** is still greater for tendon proper-derived progenitors. Biological replicates are given below each panel for each group. Statistical significance is specifically queried for PERI 7+ vs. TP 7 and PERI 45+ vs. TP 45 (Biological replicates, n = 5 to 8; Mann–Whitney-Wilcoxon test – *P* <0.01, *; *P* <0.05, #). Click here for file
