# Supplementary figures and images for: Tendon proper- and peritenon-derived progenitor cells have unique tenogenic properties
Source: Stem Cell Res Ther. 2014 Jul 8;5(4):86. doi: 10.1186/scrt475 (PMC4230637; doi:10.1186/scrt475)

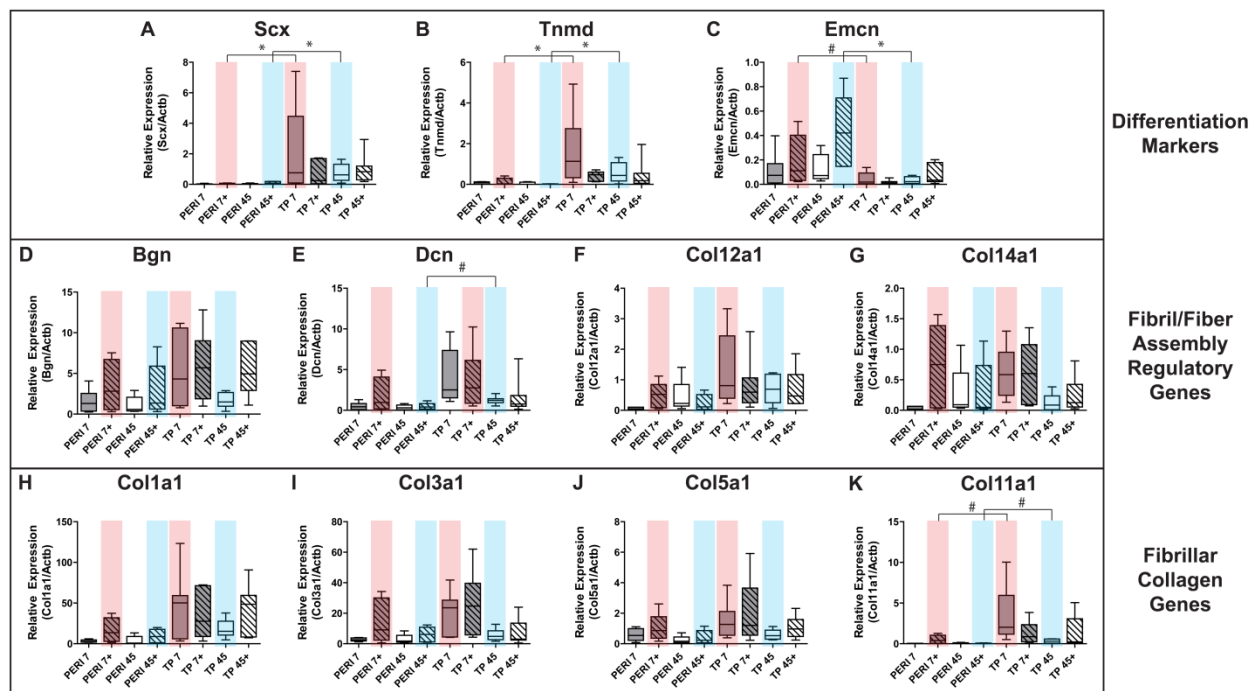

Supplement: Additional file 1: Figure S1 — GDF5 supplementation causes improved expression matrix assembly genes in peritenon progenitors seeded in constructs: an expanded view. After r-mGDF5 supplementation, expression levels for tendon markers Scx(A) and Tnmd(B) and vascular marker Emcn(C) still differed. GDF5 supplementation led to improved expression of matrix assembly genes for peritenon progenitors seeded in tendon constructs -comparing PERI + vs. TP: Day 7 (early), red boxes; Day 45 (late), blue boxes. Bolstered expression is noted for SLRPs Bgn(D) and early for Dcn(E), for FACIT collagens Col12a1(F) and Col14a1(G), and for fibril-forming collagens Col1a1(H), Col3a1(I), and Col5a1(J). Expression of Col11a1(K) is still greater for tendon proper-derived progenitors. Biological replicates are given below each panel for each group. Statistical significance is specifically queried for PERI 7+ vs. TP 7 and PERI 45+ vs. TP 45 (Biological replicates, n = 5 to 8; Mann–Whitney-Wilcoxon test – P <0.01, *; P <0.05, #). [file scrt475-S1.pdf]
